# Supplementary figures and images for: The Interactive Effect of Elevated CO2 and Herbivores on the Nitrogen-Fixing Plant Alnus incana ssp. rugosa
Source: Plants (Basel). 2021 Feb 26;10(3):440. doi: 10.3390/plants10030440 (PMC7996819; doi:10.3390/plants10030440)

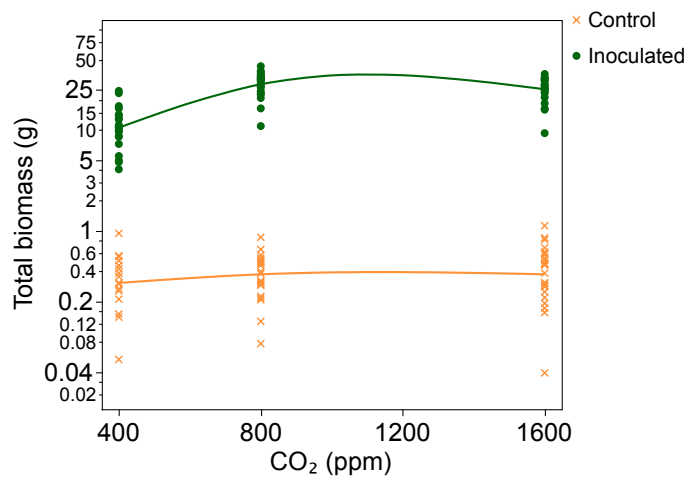

Supplement: Supplementary file 1 [file plants-10-00440-s001.zip › Figure S1.pdf]

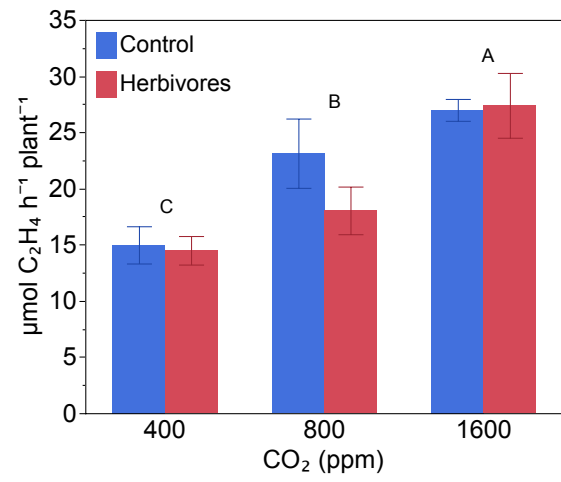

Supplement: Supplementary file 1 [file plants-10-00440-s001.zip › Figure S2.pdf]

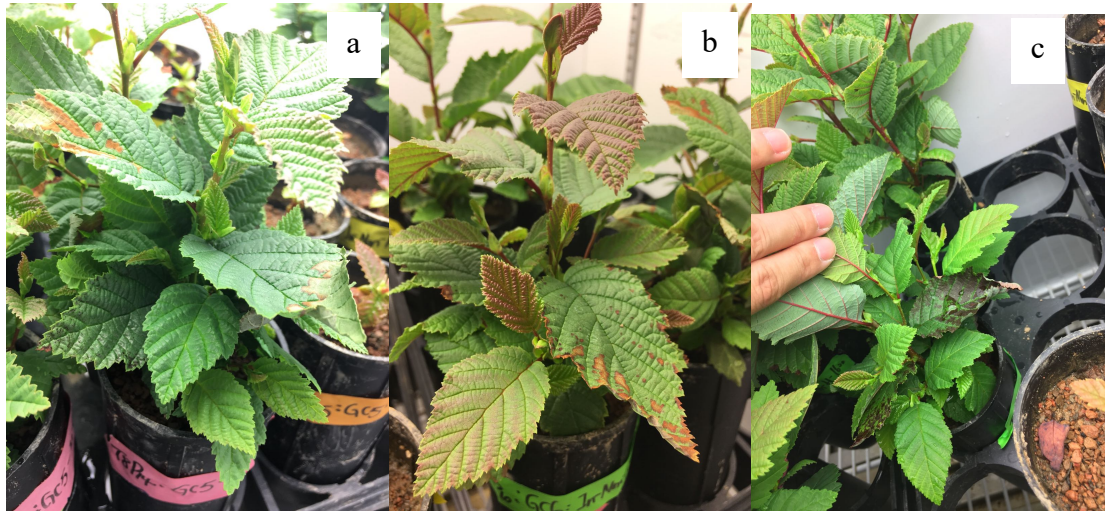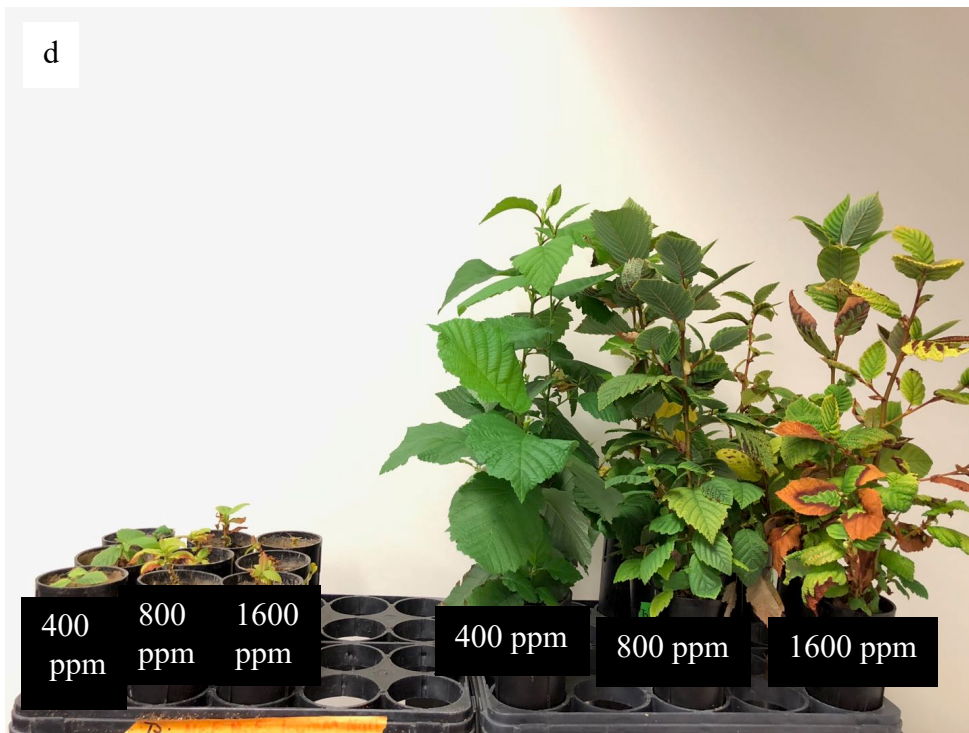

Supplement: Supplementary file 1 [file plants-10-00440-s001.zip › Figure S3.pdf]
